# Characterization of a recombinant *Aspergillus niger* GZUF36 lipase immobilized by ionic liquid modification strategy

**DOI:** 10.1007/s00253-024-13071-z

**Published:** 2024-02-24

**Authors:** Shuqi Xing, Jia Long, Wei Xie, Chaocheng Luo, Laping He, Cuiqin Li, Xuefeng Zeng

**Affiliations:** 1 Center for Research and Development of Fine Chemicals, Guizhou University, Guiyang, 550025 People’s Republic of China; 2 Key Laboratory of Agricultural and Animal Products Store & Processing of Guizhou Province, Guizhou University, Guiyang, 550025 People’s Republic of China; 3 College of Liquor and Food Engineering, Guizhou University, Guiyang, 550025 People’s Republic of China; 4 College of Chemistry and Chemical Engineering, Guizhou University, Guiyang, 550025 People’s Republic of China

**Keywords:** Lipase, Immobilization, Ionic liquid modified magnetic nanomaterials, Multipoint adsorption, Reusability

## Abstract

**Abstract:**

Enzyme immobilized on magnetic nanomaterials is a promising biocatalyst with efficient recovery under applied magnets. In this study, a recombinant extracellular lipase from *Aspergillus niger* GZUF36 (PEXANL1) expressed in *Pichia pastoris* GS115 was immobilized on ionic liquid-modified magnetic nano ferric oxide (Fe_3_O_4_@SiO_2_@ILs) via electrostatic and hydrophobic interaction. The morphology, structure, and properties of Fe_3_O_4_@SiO_2_@ILs and immobilized PEXANL1 were characterized by scanning electron microscopy, Fourier transform infrared spectroscopy, x-ray diffraction, vibration sample magnetometer, and zeta potential analysis. Under optimized conditions, the immobilization efficiency and activity recovery of immobilized PEXANL1 were 52 ± 2% and 122 ± 2%, respectively. The enzymatic properties of immobilized PEXANL1 were also investigated. The results showed that immobilized PEXANL1 achieved the maximum activity at pH 5.0 and 45 °C, and the lipolytic activity of immobilized PEXANL1 was more than twice that of PEXANL1. Compared to PEXANL1, immobilized PEXANL1 exhibited enhanced tolerance to temperature, metal ions, surfactants, and organic solvents. The operation stability experiments revealed that immobilized PEXANL1 maintained 86 ± 3% of its activity after 6 reaction cycles. The enhanced catalytic performance in enzyme immobilization on Fe_3_O_4_@SiO_2_@ILs made nanobiocatalysts a compelling choice for bio-industrial applications. Furthermore, Fe_3_O_4_@SiO_2_@ILs could also benefit various industrial enzymes and their practical uses.

**Key points:**

• *Immobilized PEXANL1 was confirmed by SEM, FT-IR, and XRD.*

• *The specific activity of immobilized PEXANL1 was more than twice that of PEXANL1.*

• *Immobilized PEXANL1 had improved properties with good operational stability.*

**Graphical abstract:**

**Figure:**
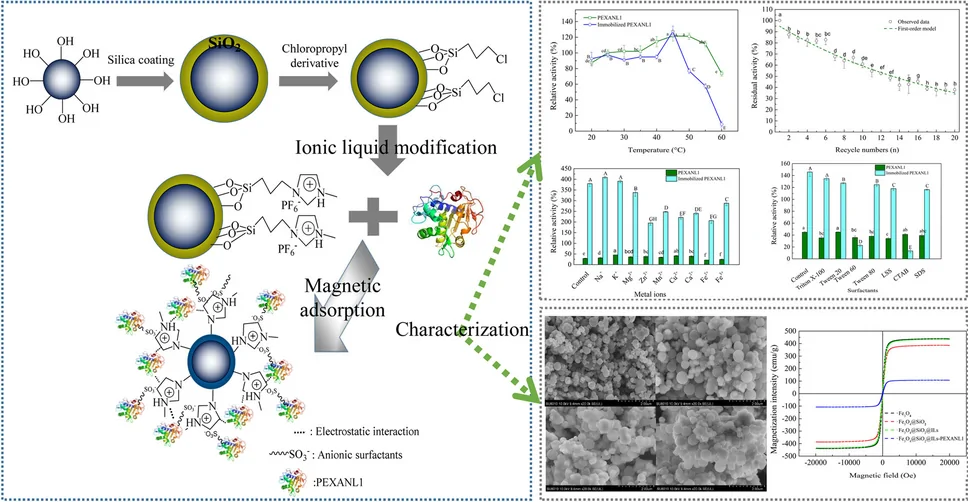

## Introduction

Lipases (EC 3.1.1.3) are a vital hydrolase that catalyzes the hydrolysis of the natural substrate lipids at the oil–water interface to produce fatty acids, glycerol, and mono- or diesters. Additionally, lipases are involved in various other reactions, including lipid synthesis, ester exchange, acidolysis, esterolysis, and enantiomeric splitting (Cai et al. 2021; Xing et al. 2021; Zhu et al. 2021). Therefore, lipases are widely used in industries such as surfactants, biodiesel, fine chemicals, wastewater treatment, pharmaceuticals, cosmetics, and flavored foods (Chen et al. 2022; Salgado et al. 2022). Unfortunately, in numerous instances, the characteristics of lipases (like other enzymes) do not meet the requirements of industrial catalysts, and their catalytic performance in industrial reactions is inadequate. Currently, there are numerous strategies to address some of the problems with lipases, using metagenomics (searching for beneficial enzymes) (Xiao et al. 2021), site-directed mutagenesis (Maldonado et al. 2023; Min et al. 2021; Wang et al. 2021a), directed evolution (Li et al. 2021), and chemical modification (Cavalcante et al. 2022). Protein engineering strategies that utilize genetic mutation based on resolved lipase crystal structures can effectively modify lipase activity and stability (Lan et al. 2021). However, lipases also face the issue of water solubility, making their recycling more complicated. Enzyme immobilization can help address this issue, and it is a tool that dramatically enhances various enzyme properties, including stability, selectivity, specificity, and inhibition (Ismail and Baek 2020). It can also be used with enzyme purification (Paul et al. 2024). However, these benefits can only be realized with carefully designed immobilization protocols.

Among the various immobilization methods, the use of magnetic nanomaterials as supports has garnered interest because of its exceptional characteristics, including low porosity, outstanding mechanical stability, and effortless separation (which can be accomplished with the use of a magnetic field) (Zhang et al. 2019). Also, as inorganic materials, magnetic nanomaterials are low-toxic and low-polluting, making them greener in chemistry. There are different types of magnetic nanomaterials, such as iron oxide (Fe_3_O_4_ and γ-Fe_2_O_3_), alloys (CoPt_3_ and FePt), pure metals (Fe and Co), spinel-type ferromagnets (MgFe_2_O_4_, MnFe_2_O_4_, and CoFe_2_O_4_). Among them, Fe_3_O_4_ magnetite nanoparticles (MNPs) have been used to immobilize oxidoreductases, hydrolases, or transferases, enhancing their reusability, storage stability, temperature stability, and enzyme activity (Bezerra et al. 2020; Dong et al. 2022).

Although Fe_3_O_4_ MNPs have many advantages, bare ferromagnetic nanoparticles are prone to oxidation, instability under acidic conditions, and magnetic dipole gravity. Additionally, their large surface area-to-volume ratio makes magnetic carriers likely to aggregate, so surface modification of Fe_3_O_4_ MNPs through amination, carboxylation, or thiolation is typically necessary. Surface modification of Fe_3_O_4_ MNPs offers several advantages. Firstly, covering the bare magnetic surface with inert materials improves the stability of Fe_3_O_4_ MNPs. Secondly, modifying the aldehyde, amine, diimine, carboxyl, or hydroxyl groups on Fe_3_O_4_ MNPs allows for the combination of magnetic carriers with enzymes, which can be achieved using bifunctional molecules, polymers, or dendritic macromolecules. This modification helps to capture enzymes in the solution. Finally, surface modification adjusts the hydrophobic and hydrophilic balance of the carrier to make the enzyme, carrier, and substrate more compatible.

Ionic liquids (ILs) are molten salts with low melting points, strong thermal stability, and exceptional solubility, making them potentially useful in catalysis, extraction, and absorption. (Kaur et al. 2022; Mokhodoeva et al. 2021). Modifying the surface of magnetic nanoparticle carriers with ILs would improve the electrostatic interaction, hydrophobic interaction, and hydrogen bond between the carriers and the lipase, efficiently preventing the leakage of lipase (Qin et al. 2016). The introduction of ILs increases hydrophobic interaction between the carriers and the lipase, leading to the opening of the lipase lid and making its active site more accessible, and the activity of the lipase is enhanced as a result of interface activation (Brzozowski et al. 1991; Mathesh et al. 2016; Wang et al. 2021b). Moreover, the enhancement of the activity of lipase immobilization by ILs may also be attributed to the increase in the rigidity of the lipase structure (Jiang et al. 2009). From these perspectives, the IL-modified magnetic nanoparticle carriers have great potential for immobilizing lipase. This surface modification can improve the surface properties for the activity and stability of the lipase.

In this research, we prepared the ionic liquid-modified Fe_3_O_4_ MNPs and utilized them to immobilize a recombinant lipase from *A. niger* GZUF36 (PEXANL1). We investigated the optimization of the immobilization process and evaluated the enzymatic properties of the immobilized PEXANL1.

## Materials and methods

### Materials

The engineering strain *P. pastoris* GS115 producing the recombinant lipase from *A. niger* GZUF36 was prepared in our previous work (Xing et al. 2021) and stored in our laboratory. Fe_3_O_4_, tetraethyl orthosilicate (TEOS), 3-chloropropyltrimethoxysilane (CP-TMS), N-methylimidazole (MI), and *p*-nitrophenyl palmitate (*p*-NPP) were purchased from Shanghai Macklin Biochemical Technology Co., Ltd. The BCA assay and standard bovine serum albumin (BSA) were obtained from Beijing Solarbio Science & Technology Co., Ltd. Potassium hexafluorophosphate (KPF_6_) and tetraethyl orthosilicate were purchased from Shanghai Yien Chemical Technology Co., Ltd. All other reagents were analytical grade unless otherwise specified.

### Production and purification of PEXANL1

The production and purification of PEXANL1 were carried out following the methods outlined in our previous study (Xing et al. 2021). The purified PEXANL1 was stored in a 20 mM Tris buffer (pH 8.0) and used for all immobilization studies.

### Preparation of magnetic nanoparticles modified with ionic liquids (Fe_3_O_4_@SiO_2_@ILs)

Fe_3_O_4_@SiO_2_@ILs were prepared according to the procedures outlined by Latifeh et al. (2016) and Yamini et al. (2017) with minor adjustments and involved four well-defined steps. First, Fe_3_O_4_ was coated with silica. Twelve grams of nano-size Fe_3_O_4_ was added to 800 mL of 2-propanol and 100 mL of ultrapure water and sonicated for 15 min to achieve good dispersion. Eighty milliliters (25 wt%) of ammonium hydroxide and 40 mL tetraethyl orthosilicate were added, and the mixture was magnetically stirred at 30 ℃ for 12 h. It should be noted that the reaction temperature should be higher than 20 ℃ for SiO_2_ to cover Fe_3_O_4_ adequately. Subsequently, the synthesized Fe_3_O_4_@SiO_2_ was recovered by an external magnet, washed alternately with deionized water and absolute ethanol, and dried at 80 ℃ for use.

In the second step, 5.0 g of Fe_3_O_4_@SiO_2_ was placed in 600 mL dried toluene, and then 5.0 mL 3-chloropropyltrimethoxysilane (CP-TMS) and 0.5 mL triethylamine were added. The mixture was then refluxed at 55 ℃ for 48 h. The resulting chloropropyl-derivatized product (Fe_3_O_4_@SiO_2_@CP-TMS) was collected using an external magnet and then washed alternately with 2-propanol, absolute ethanol, and deionized water to remove any oily toluene on the surface. Finally, it was dried at 80 °C for later use.

In the third step, Fe_3_O_4_@SiO_2_@Cl was modified with N-methylimidazole (MI). Five grams Fe_3_O_4_@SiO_2_@CP-TMS was redispersed in 600 mL dried benzene, and 5.0 mL N-methylimidazole was added, and refluxed at 55 ℃ for 48 h. After the refluxing was completed, the obtained methylimidazole-chloride functionalized magnetic nano-Fe_3_O_4_ (Fe_3_O_4_@SiO_2_@CP-TMS@MI) was recovered by an external magnet, washed alternately with 2-propanol, absolute ethanol, and deionized water and dried at 80℃ for later use.

Finally, the methylimidazole-hexafluorophosphate functionalized magnetic nano-Fe_3_O_4_ (Fe_3_O_4_@SiO_2_@ILs) was obtained through a simple ion exchange reaction with hexafluorophosphate. The specific experimental procedure was as follows: 1 g of Fe_3_O_4_@SiO_2_@CP-TMS@MI was added to 100 mL of 7% (w/v) KPF_6_ aqueous solution and stirred twice at 30 ℃ for 2 h each time. The resulting Fe_3_O_4_@SiO_2_@ILs were separated by an external magnet, washed with deionized water, and dried.

### Immobilization of PEXANL1

The immobilization of PEXANL1 was performed by mixing 10 mg Fe_3_O_4_@SiO_2_@ILs, 4 mL purified PEXANL1 (protein concentration, 0.2 mg/mL), and 1 mL anionic surfactant of the appropriate concentration. The mixture was then sonicated for 30 s and shaken at a stirring speed of 150 rpm at 30 ℃ for 4 h. After the reaction, the immobilized PEXANL1 (Fe_3_O_4_@SiO_2_@IL-PEXANL1) was separated by an external magnet and was washed twice with 5 mL of sodium dihydrogen phosphate-citrate buffer solution (pH 8.0) to remove unbound PEXANL1. Subsequently, Fe_3_O_4_@SiO_2_@IL-PEXANL1 was dried and stored at 25 ℃ for further analysis.

Optimization of immobilization was conducted to maximize the enzyme activity recovery and immobilization efficiency of Fe_3_O_4_@SiO_2_@IL-PEXANL1. Factors and levels include different surfactant types (sodium dodecyl sulfate (SDS), alkyl polyglycoside (APG), sodium methyl cocoyl taurate (AK), potassium lauryl phosphate (MAP), sodium 2-(nonanoyloxy)ethanesulfonate (SCL), fatty acid methyl ester sulfonate (MES), and sodium bis (2-ethylhexylpolyoxyalkylene) sulfosuccinates (AOT)), SDS concentration (0.005%, 0.01%, 0.015%, 0.02%, 0.025%, and 0.03%), pH (3.0, 4.0, 5.0, 6.0, 7.0, and 8.0), carrier addition amount (5, 10, 15, 20, 25, and 30 mg), immobilization time (1, 2, 3, 4, 5, and 6 h) and PEXANL1 protein concentration (0.1, 0.2, 0.3, 0.4, 0.5, and 0.6 mg/mL).

### Lipase activity assay

The enzyme activity was measured using the method outlined by Zhang et al. (2020) with some minor adjustments. In a typical reaction, the reaction mixture included 1.8 mL of 0.05 M sodium dihydrogen phosphate-citrate buffer (pH 5.0), 0.1 mL of 10 mM *p*-NPP (dissolved in 2-propanol by ultrasonic treatment), and 0.1 mL of appropriately diluted lipase solution or 10 mg of the immobilized enzyme. The reaction was conducted at 45 °C for 10 min, and then the mixture was added 0.5 mL of 15% (w/v) trichloroacetic acid to terminate the reaction. The resulting mixture was supplemented with 0.5 mL 10% (w/v) Na_2_CO_3_ to facilitate color development and then diluted 5 times with a 20 mM Tris–HCl buffer solution (pH 8.0). The *p*-nitrophenol (*p*-NP) released from the enzymatic reaction was measured spectrophotometrically at 405 nm. The absorbance of 0.1–1.0 mM *p*-NP at 405 nm was measured, and a standard curve was made. One unit (U) of enzyme activity was defined as the amount of enzyme required that releases 1 µmol of *p*-NP per minute under test conditions. The activity recovery and the immobilization efficiency were used to evaluate the performance of immobilization PEXANL1 and were calculated using the following Eqs. (1) and (2), respectively (Zhao et al. 2022):

1 $$\text{Enzyme activity recovery}\;\left(\%\right)=\frac{\text{Total enzyme activity of immobilized enzyme (U)}}{\text{Total enzyme activity of used free enzyme (U)}}\times {100}$$

2 $$\mathrm{Immobilization\;efficiency }\;\left(\%\right)=\frac{{c}_{1}{v}_{1}-{c}_{2}{v}_{2}}{{c}_{1}{v}_{1}}\times 100$$

where *c*_1_ and *c*_2_ represent the concentration of enzyme initially used for immobilization (mg/mL) and the unbound enzyme in the immobilization process (mg/mL), respectively; *v*_1_ and *v*_2_ denote the volume of enzyme initially used for immobilization (mL) and the unbound enzyme in the immobilization process (mL), respectively. The BCA assay (Smith et al. 1985) determined the enzyme concentration using bovine serum albumin (BSA) as a standard. The concentration of unbound enzyme was determined by measuring the supernatant after immobilization.

### Characterization of the synthesized ionic liquid-modified magnetic nanoparticle carriers

The size and surface morphology of Fe_3_O_4_, Fe_3_O_4_@SiO_2_, Fe_3_O_4_@SiO_2_@ILs, and Fe_3_O_4_@SiO_2_@IL-PEXANL1 were observed using a scanning electron microscope (SEM) (Hitachi, HITACHISU8010).

Fourier-transform infrared (FT-IR) spectra of the samples (KBr pellet) were obtained from a Nicolet iS5 INFRARED FT-IR spectroscopy to analyze the presence of functional groups in Fe_3_O_4_, Fe_3_O_4_@SiO_2_, Fe_3_O_4_@SiO_2_@CP-TMS, Fe_3_O_4_@SiO_2_@ILs, and Fe_3_O_4_@SiO_2_@IL-PEXANL1.

X-ray diffraction patterns (XRD) of all the samples were collected from an Empyrean PANalytical B.V. X-ray diffractometer to analyze the composition and crystallographic structure of Fe_3_O_4_, Fe_3_O_4_@SiO_2_, Fe_3_O_4_@SiO_2_@CP-TMS, and Fe_3_O_4_@SiO_2_@ILs.

The hysteresis lines of the samples were obtained using a vibrating sample magnetometer (VSM) (Lakeshore 7040 ± 2 T, USA), with a magnetic field amplitude of ± 20 k Oe at room temperature to determine the magnetic characterization of Fe_3_O_4_, Fe_3_O_4_@SiO_2_, Fe_3_O_4_@SiO_2_@CP-TMS, and Fe_3_O_4_@SiO_2_@ILs.

The zeta potential (ζ) values of Fe_3_O_4_, Fe_3_O_4_@SiO_2_, Fe_3_O_4_@SiO_2_@CP-TMS, Fe_3_O_4_@SiO_2_@ILs, and Fe_3_O_4_@SiO_2_@IL-PEXANL1 in a 20 mM Tri-HCl buffer solution (pH 7.0) were measured using a Zeta potential analyzer (DelsaNanoC, Beckman Coulter, USA).

### Effect of temperature and pH on PEXANL1 and immobilized PEXANL1

The optimal temperature of PEXANL1 and immobilized PEXANL1 was studied by measuring their enzyme activity within a temperature range of 20 °C to 60 °C, with an interval of 5 °C. The optimal pH of PEXANL1 and immobilized PEXANL1 was carried out by measuring their enzyme activity in various pH levels (pH 2.0–8.0 using 50 mM disodium hydrogen phosphate-citrate buffer and pH 9.0–10.0 using 50 mM sodium bicarbonate-sodium bicarbonate buffer), as described in the lipase activity assay in the “Materials and methods” section. The enzyme activity was expressed as the relative enzyme activity (%), and the activity of PEXANL1 under the optimum conditions (45 °C and pH 5.0) was considered 100%. Relative enzyme activity in varying conditions was then calculated against this standard.

The thermal stability of PEXANL1 and immobilized PEXANL1 was assessed by incubating the enzyme within a temperature range of 20 to 60 °C, in 5 °C increments, for 1 h. After incubation, the residual activity was determined under the optimum conditions (45 °C and pH 5.0) using the lipase activity assay described in the “Materials and methods” section.

Similarly, the pH stability of PEXANL1 and immobilized PEXANL1 was performed by incubating the enzyme in different pH levels (pH 2.0–8.0 using 50 mM disodium hydrogen phosphate-citrate buffer and pH 9.0–10.0 using 50 mM sodium bicarbonate-sodium bicarbonate buffer) at 4 °C for 48 h. Subsequently, the residual activity was determined under the optimum conditions (45 °C and pH 5.0) as according to the lipase activity assay described in the “Materials and methods” section previously.

### Determination of the operational stability of immobilized PEXANL1

The operational stability of immobilized PEXANL1 was assessed through a hydrolysis reaction of *p*-NPP under the optimal conditions (45 °C and pH 5.0), over 20 consecutive cycles, with slight modifications from our prior study (Zhu et al. 2021). Following each 10-min reaction cycle, the immobilized PEXANL1 was separated using a magnetic field, then rinsed with 50 mM sodium dihydrophosphate citrate buffer (pH 5.0) several times until the residual substrate from the magnetic nanoparticles was removed. The activity of the immobilized PEXANL1 in the first cycle was defined as 100%. According to the literature (Mota et al. 2020), the change in enzyme activity behavior was analyzed by minimizing the residual sum of squares between test points and first-order inactivation models using Origin 9.0.

### Effect of metal ions on PEXANL1 and immobilized PEXANL1

The stability of PEXANL1 and immobilized PEXANL1 in the presence of metal ions was evaluated by measuring their residual activity after incubation with various metal ions with a final concentration of 5 mM at room temperature for 2 h. The residual activity was determined under the optimal conditions (45 °C and pH 5.0), and the results were expressed as relative activity (%). For immobilized PEXANL1, it was resuspended in a 20 mM Tris–HCl buffer at pH 8.0 and then mixed with different metal ion solutions (the final concentration of 5 mM). PEXANL1 and immobilized PEXANL1 were also tested without metal ion treatment to serve as controls.

### Effect of surfactants and organic solvents on PEXANL1 and immobilized PEXANL1

The effect of surfactants on PEXANL1 and immobilized PEXANL1 was assessed by measuring their residual activity after incubation with various surfactants with a final concentration of 0.01% (w/v) at room temperature for 2 h. The residual activity was determined under the optimal conditions (45 °C and pH 5.0) and was expressed as relative activity (%). The surfactants tested included non-ionic (TritonX-100, Tween 20, Tween 60, and Tween 80), cationic surfactants (hexadecyltrimethylammonium bromide, CTAB), and anionic surfactants (lauroylsarcosine sodium salt (LSS) and sodium dodecyl sulfate (SDS)). The control was the enzyme activity of PEXANL1 or immobilized PEXANL1 without surfactant incubation.

The effect of organic solvents on PEXANL1 and immobilized PEXANL was performed by measuring their residual activity after incubation with various organic solvents at room temperature for 2 h. The organic solvents tested included diethyl ether, tetrahydrofuran, xylene, isopropanol, dimethyl sulfoxide, methanol, glacial acetic acid, dichloromethane, hexane, tert-butanol, acetone, and acetonitrile. The enzyme used here was lyophilized powder obtained using a vacuum freeze dryer (FD-1A-50 plus, Biocool, Beijing, China). For immobilized PEXANL1, after incubation with organic solvents, it was separated using an external magnet, dried at room temperature, and resuspended in 2 mL of 50 mM citrate disodium hydrogen phosphate buffer at pH 8.0. The residual activity of PEXANL1 or immobilized PEXANL1 treated with organic solvent was determined under the optimal conditions (45 °C and pH 5.0) as described in the lipase activity assay in the “Materials and methods” section. PEXANL1 or immobilized PEXANL1 without organic solvent treatment was used as the control.

### Determination of kinetic parameters of PEXANL1 and immobilized PEXANL1

The kinetic parameters, Michaelis constant (*K*_m_), and maximum velocity (*V*_max_) of PEXANL1 and immobilized PEXANL1 were determined by measuring enzyme activity across a range of substrate concentrations from 5 to 30 mM under the optimal conditions (45 °C and pH 5.0). According to the curve fitting of the reciprocal plot of reaction rate versus substrate concentration, *K*_m_ and *V*_max_ were calculated using the Lineweaver–Burk plot (Rodriguez et al. 2019).

### Determination of sn-1, 3 specificity of PEXANL1 and immobilized PEXANL1

The sn-1, 3 specificity of PEXANL1 and immobilized PEXANL1 were studied according to our previous report (Xing et al. 2021). In brief, PEXANL1 and immobilized PEXANL1 were employed to catalyze the esterification of glycerol and oleic acid in a solvent-free system, respectively. The reaction medium consisted of glycerol:oleic acid of 2:1 molar ratio, 10% enzyme loading by substrate weight, and 0.6% water content. The reaction mixture was processed for 5 min through ultrasound and stirred under magnetism at 300 r/min for 10 h at 30 °C. At the end of the reaction, the mixture was extracted using hexane, and the product composition (sn-1, 3-diacylglycerol and sn-1, 2-diacylglycerol) and sn-1, 3 selectivity were quantified and assessed by our previous report (Xing et al. 2021). The sn-1, 3 selectivity of lipase was calculated using the following equation:

3 $${\mathrm{Sn}}-1,\;3\mathrm{\;specificity\;}(\mathrm{\%}) = \frac{{m}_{sn-1,\;3-DAG}}{{m}_{sn-1,\;3-DAG}+{m}_{sn-1,\;2-DAG}}$$

where m_sn-1, 3-DAG_ and m_sn-1, 2-DAG_ are the amounts of sn-1, 3-diacylglycerol and sn-1, 2-diacylglycerol (mg), respectively.

### Statistical analysis

All experiments above were repeated three times, and the results were expressed as mean ± standard deviation. The significant difference analysis was performed using SPSS (IBM 22.0).

## Results

### Characterization of the synthesized ionic liquid-modified magnetic nanoparticle carriers and immobilization processes

**Fig. 1:**
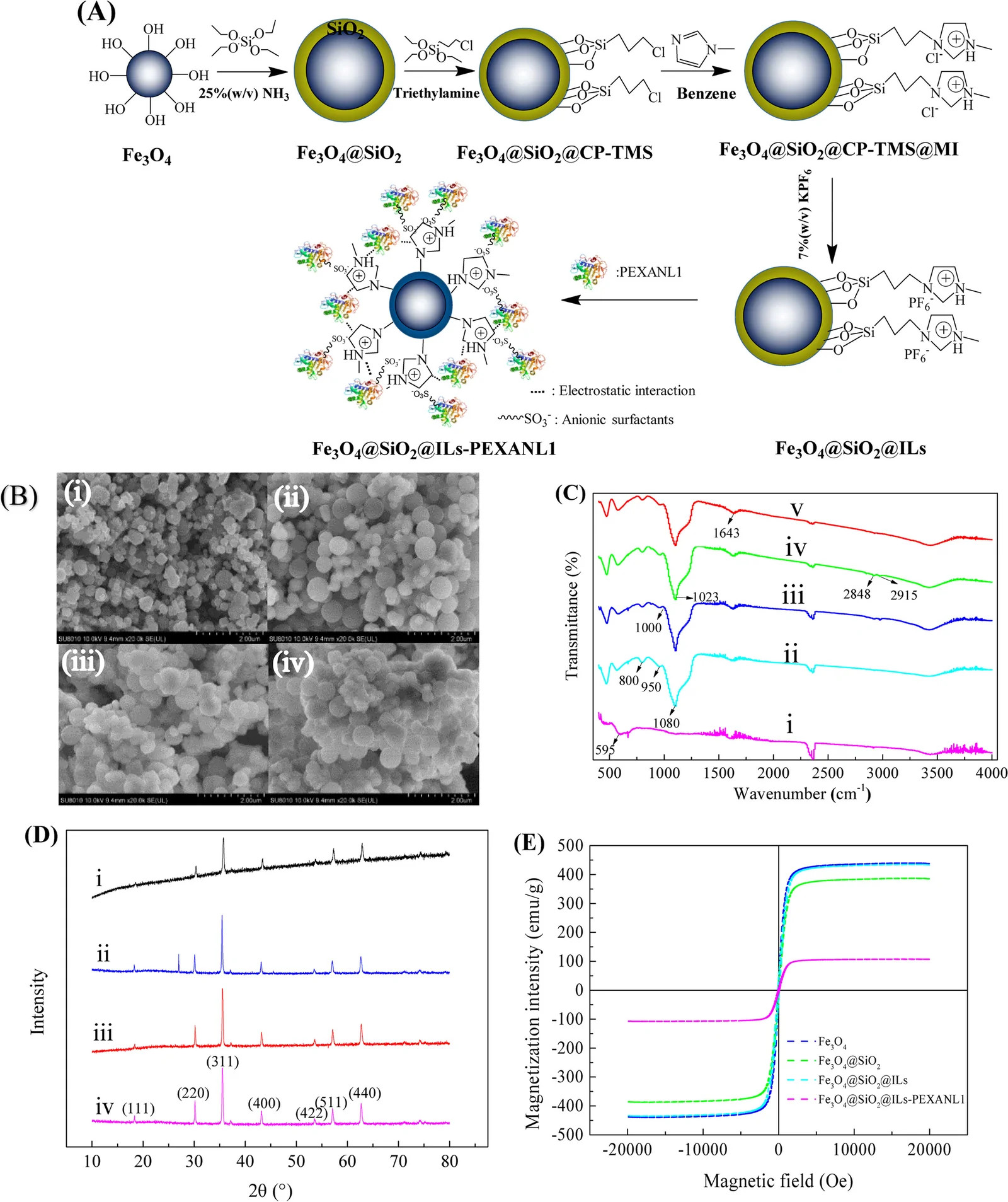
Characterization of the synthesized ionic liquid modified magnetic nanoparticle carriers and immobilization processes. **A** Schematic diagram of PEXANL1 immobilization onto Fe_3_O_4_@SiO_2_@ILs nanocarrier; **B** SEM images of (i) Fe_3_O_4_, (ii) Fe_3_O_4_@SiO_2_, (iii) Fe_3_O_4_@SiO_2_@ILs, and (iv) Fe_3_O_4_@SiO_2_@IL-PEXANL1; **C** The FT-IR spectra of (i) Fe_3_O_4_, (ii) Fe_3_O_4_@SiO_2_, (iii) Fe_3_O_4_@SiO_2_@CP-TMS, (iv) Fe_3_O_4_@SiO_2_@ILs, and (v) Fe_3_O_4_@SiO_2_@IL-PEXANL1; **D** the XRD spectra of (i) Fe_3_O_4_, (ii) Fe_3_O_4_@SiO_2_, (iii) Fe_3_O_4_@SiO_2_@CP-TMS, and (iv) Fe_3_O_4_@SiO_2_@ILs; **E** magnetic hysteresis loops of Fe_3_O_4_, Fe_3_O_4_@SiO_2_, Fe_3_O_4_@SiO_2_@ILs, and Fe_3_O_4_@SiO_2_@IL-PEXANL1

The schematic diagram in Fig. 1(A) illustrates the step-by-step approach to preparing ionic liquid-modified magnetic nanoparticles to immobilize PEXANL1. The magnetic nanoparticles during immobilization processes were characterized by SEM, FT-IR, XRD, VSM, and Zeta potentials, as shown in Fig. 1(B–E).

### SEM

The size and surface morphology of Fe_3_O_4_, Fe_3_O_4_@SiO_2_, Fe_3_O_4_@SiO_2_@ILs, and immobilized PEXANL1 were observed through SEM. As shown in Fig. 1(B(i)), Fe_3_O_4_ was spherical particles with uneven size distribution and rough surface. After SiO_2_ modification, the resulting Fe_3_O_4_@SiO_2_ was a regular spherical with shape, uniform distribution, larger particle size, and dispersed particles (Fig. 1(B(ii))). The morphology of Fe_3_O_4_@SiO_2_@ILs is shown in Fig. 1(B(iii)). After modification by the ionic liquid, Fe_3_O_4_@SiO_2_@ILs was smoother and more uniform with small aggregation. After PEXANL1 was fixed by Fe_3_O_4_@SiO_2_@ILs, the particle size did not change significantly (Fig. 1(B(iv))).

### FT-IR

As it is shown in Fig. 1(C(i)), the characteristic absorption peaks between 575 and 600 cm^−1^ correspond to Fe–O bond vibration (Li et al. 2015), indicating the presence of Fe_3_O_4_. The FT-IR spectra of Fe_3_O_4_@SiO_2_ showed characteristic absorption peaks around 1080 cm^−1^, 960 cm^−1^, and 800 cm^−1^ (Fig. 1(C(ii))), which were generated by Si–O–Si antisymmetric stretching, Si–O symmetric, and Si–O–Si symmetric modes, respectively (Garkoti et al. 2017), indicating that the surface modification of Fe_3_O_4_ has been successfully performed using silica. As it is shown in Fig. 1(C(iii)), the FT-IR spectra of Fe_3_O_4_@SiO_2_@CP-TMS showed the characteristic absorption peak at around 1000 cm^−1^, which was attributed to N–H stretching, confirming the existence of CP-TMS. As it is shown in Fig. 1(C(iv)), the FT-IR spectra of Fe_3_O_4_@SiO_2_@ILs showed specific absorption peaks near 2915 cm^−1^, 2848 cm^−1^, and 1023 cm^−1^ due to the presence of imidazole ring (Medeiros et al. 2012). In addition, the peak pattern became sharper at 1623 cm^−1^. These results indicated that Fe_3_O_4_@SiO_2_@ILs was successfully prepared. Furthermore, there were new characteristic absorption peaks in 1600–1700 cm^−1^ (Fig. 1(C(v))) due to C = O–N–H groups stretching, indicating that PEXANL1 was immobilized onto Fe_3_O_4_@SiO_2_@ILs carrier.

### XRD

The XRD pattern of Fe_3_O_4_ showed characteristic peaks at the corresponding 2θ angles (Fig. 1(D(i))), whose characteristic peaks mainly appeared at 2θ = 18.32°, 30.42°, 35.86°, 43.51°, 54.03°, 57.49°, and 63.37°, corresponding to the crystallographic planes of (111), (220), (311), ( 400), (422), (511), and (440) (Liu et al. 2018). The XRD pattern of Fe_3_O_4_@SiO_2_ is shown in Fig. 1(D(ii)). A new peak appearing at 2θ = 26.6° was produced by the amorphous silica shell on the surface of Fe_3_O_4_@SiO_2_ (Nadar and Rathod 2018). In contrast, CP-MES and ionic liquid modification did not change the crystal structure of Fe_3_O_4_@SiO_2_ (Fig. 1(D(iii) (iv))).

### VSM

As it is shown in Fig. 1(E), the magnetic hysteresis curves of the magnetic carrier and the immobilized enzyme were S-shaped, and both passed through the “0” point, indicating that the magnetic carrier had no remaining magnetic and coercive force. The calculated results showed that magnetic strengths of Fe_3_O_4_, Fe_3_O_4_@SiO_2_, Fe_3_O_4_@SiO_2_@ILs, and immobilized PEXANL1 were 439.59 emu/g, 386.65 emu/g, 435.17 emu/g, and 107.55 emu/g, respectively.

### Zeta potentials

The zeta potentials of PEXANL1, Fe_3_O_4_, Fe_3_O_4_@SiO_2_, Fe_3_O_4_@SiO_2_@CP-TMS, Fe_3_O_4_@SiO_2_@ILs, and immobilized PEXANL1 are shown in Table 1. The zeta potential value of PEXANL1 in pH 7.0 was − 18.48 ± 0.81, and that of Fe_3_O_4_ in pH 7.0 was − 12.33 ± 1.48. The zeta potential of Fe_3_O_4_@SiO_2_ in pH 7.0 (− 49.17 ± 1.77) was much smaller than that of Fe_3_O_4_ in pH 7.0, while the zeta potential value of Fe_3_O_4_@SiO_2_@CP-TMS in pH 7.0 (42.52 ± 2.26) was significantly increased after the chloropropyl derivatization of Fe_3_O_4_@SiO_2_. It was observed that the zeta potential values of Fe_3_O_4_@SiO_2_@ILs in pH 7.0 dropped to 25.05 ± 1.85. However, the zeta potential values of PEXANL1 immobilized on Fe_3_O_4_@SiO_2_@ILs in pH 7.0 was − 34.04 ± 1.00, significantly decreasing.

**Table 1:** Zeta potential of magnetic immobilized carriers and immobilized enzymes

Magnetic immobilized carriers/immobilized enzymes	Zeta potential
PEXANL	− 18.48 ± 0.81
Fe_3_O_4_	− 12.33 ± 1.48
Fe_3_O_4_@SiO_2_	− 49.17 ± 1.77
Fe_3_O_4_@SiO_2_@CP-TMS	42.52 ± 2.26
Fe_3_O_4_@SiO_2_@ILs	25.05 ± 1.85
Fe_3_O_4_@SiO_2_@IL-PEXANL1	− 34.04 ± 1.00

### Optimizing conditions for PEXANL1 immobilization on ionic liquid-modified magnetic nanoparticle

**Fig. 2:**
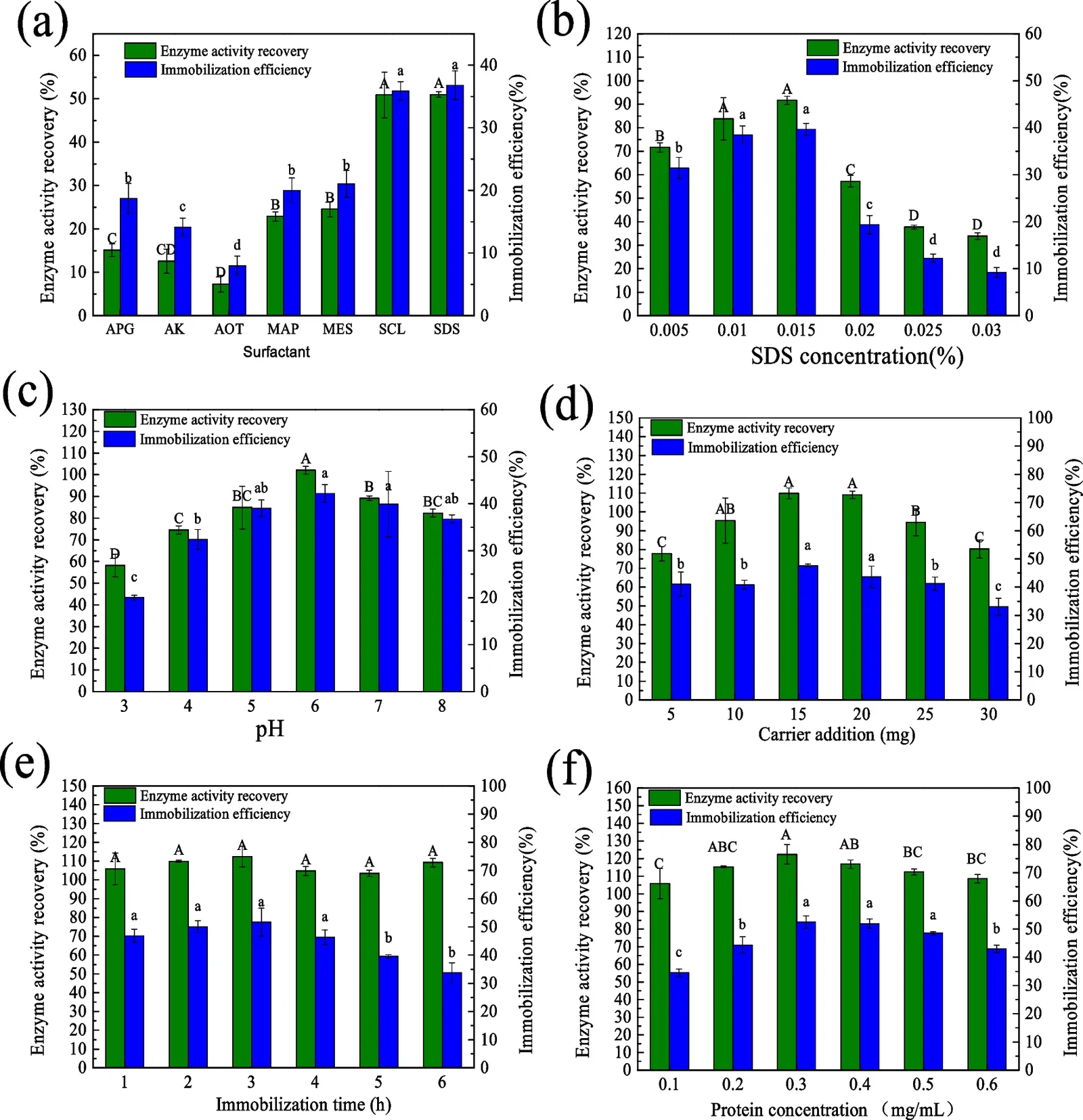
Optimized immobilization conditions of (**a**) anionic surfactant type, including sodium dodecyl sulfate (SDS), alkyl polyglycoside (APG), sodium methyl cocoyl taurate (AK), potassium lauryl phosphate (MAP), sodium 2-(nonanoyloxy) ethanesulfonate (SCL), fatty acid methyl ester sulfonate (MES), and sodium bis (2-ethylhexylpolyoxyalkylene) sulfosuccinates (AOT). (**b**) SDS concentration, (**c**) pH, (**d**) carrier addition amount, (**e**) immobilization time, and (**f**) protein concentration. Different lowercase or uppercase letters of the same bar chart in the same subgraph indicate significant differences (*p* < 0.05)

The immobilization conditions including anionic surfactant type and content, pH, carrier addition content, immobilization time, and enzyme concentration were studied. The optimized factors and levels are detailed in Table 2, and the detailed changes in immobilization efficiency and enzyme activity recovery are shown in Fig. 2.

**Table 2:** Effects of immobilization parameters on the immobilization efficiency and activity recovery

Factors	Optimized range	Optimum value	Activity recovery (%)	Immobilization efficiency (%)
SDS concentration (%, wt)	0.05–0.03	0.015	92 ± 2	40 ± 1
pH	3.0–8.0	6.0	102 ± 2	42 ± 2
Carrier addition amount (mg)	5–30	15	110 ± 3	48 ± 1
Immobilization time (h)	1–6	3	112 ± 5	52 ± 5
Enzyme concentration (mg/mL)	0.1–0.6	0.3	122 ± 2	52 ± 2

As shown in Fig. 2(a), the activity recovery of immobilized PEXANL1 was the highest when using SDS (*p* < 0.05). Conversely, with SCL, APG, AK, MAP-K, and MES, the activity recovery was significantly reduced compared to SDS. The lowest activity recovery was observed with AOT. These results indicated SDS was the optimal anionic surfactant, achieving an activity recovery and immobilization efficiency of 50.97% and 36.79%, respectively (Fig. 2(a)).

The impact of varying SDS concentrations on lipase immobilization was investigated. It was found that the optimal SDS concentration was 0.015%, yielding an enzyme activity recovery and immobilization efficiency of 91.67% and 39.66%, respectively (Fig. 2(b)). Below this concentration, the immobilization efficiency and activity recovery were slightly reduced due to insufficient micelle formation between the carrier and the enzyme. Conversely, excessive SDS significantly reduced immobilization efficiency and activity recovery (*p* < 0.05).

Immobilization efficiency and activity recovery were enhanced in a neutral, acidic environment (Fig. 2(c)), as pH affects the charges’ properties and amounts on both enzyme and carrier, dramatically influencing the immobilization. Consequently, the activity recovery and immobilization efficiency increased rapidly initially and showed the highest values (102.07% and 42.16%, respectively) at pH 6.0.

The effect of varying Fe_3_O_4_@SiO_2_@ILs amounts is shown in Fig. 2(d). As the amount of Fe_3_O_4_@SiO_2_@ILs increased, the activity recovery and immobilization efficiency improved. It was found that the optimal amount of Fe_3_O_4_@SiO_2_@ILs was 15 mg or 20 mg, yielding an activity recovery of 109.87% and 109.10%, and immobilization efficiency of 40.84% and 43.61%, respectively, with no significant difference (*p* < 0.05). However, further increases in carrier content negatively impacted the mass transfer of PEXANL1 (Sigurdardóttir et al. 2018). Thus, the activity recovery and immobilization efficiency were reduced.

Immobilization time had no significant effect on the activity recovery (*p* > 0.05), while immobilization efficiency slightly decreased with increasing time (Fig. 2(e)). The reason why immobilization efficiency decreased as increasing time may be due to too many carriers would limit the binding to lipase, leading to a decrease in immobilization efficiency (Zhao et al. 2019). So, the optimal immobilization time was 3 h.

For enzyme concentration, six different PEXANL1 concentrations (0.1, 0.2, 0.3, 0.4, 0.5, and 0.6 mg/mL) were immobilized onto 15-mg support in phosphate buffer (pH 6.0) to determine the proper loading. The activity recovery (122.37%) and immobilization efficiency (52.46%) of the immobilized enzyme reached the maximum when the PEXANL1 concentration was 0.3 mg/mL. However, the activity recovery and immobilization efficiency decreased with the continued increase of PEXANL1 concentrations (Fig. 2(f)). So, a PEXANL1 enzyme concentration of 0.3 mg/mL was chosen for the most appropriate immobilization process.

### Optimum temperature and temperature stability

**Fig. 3:**
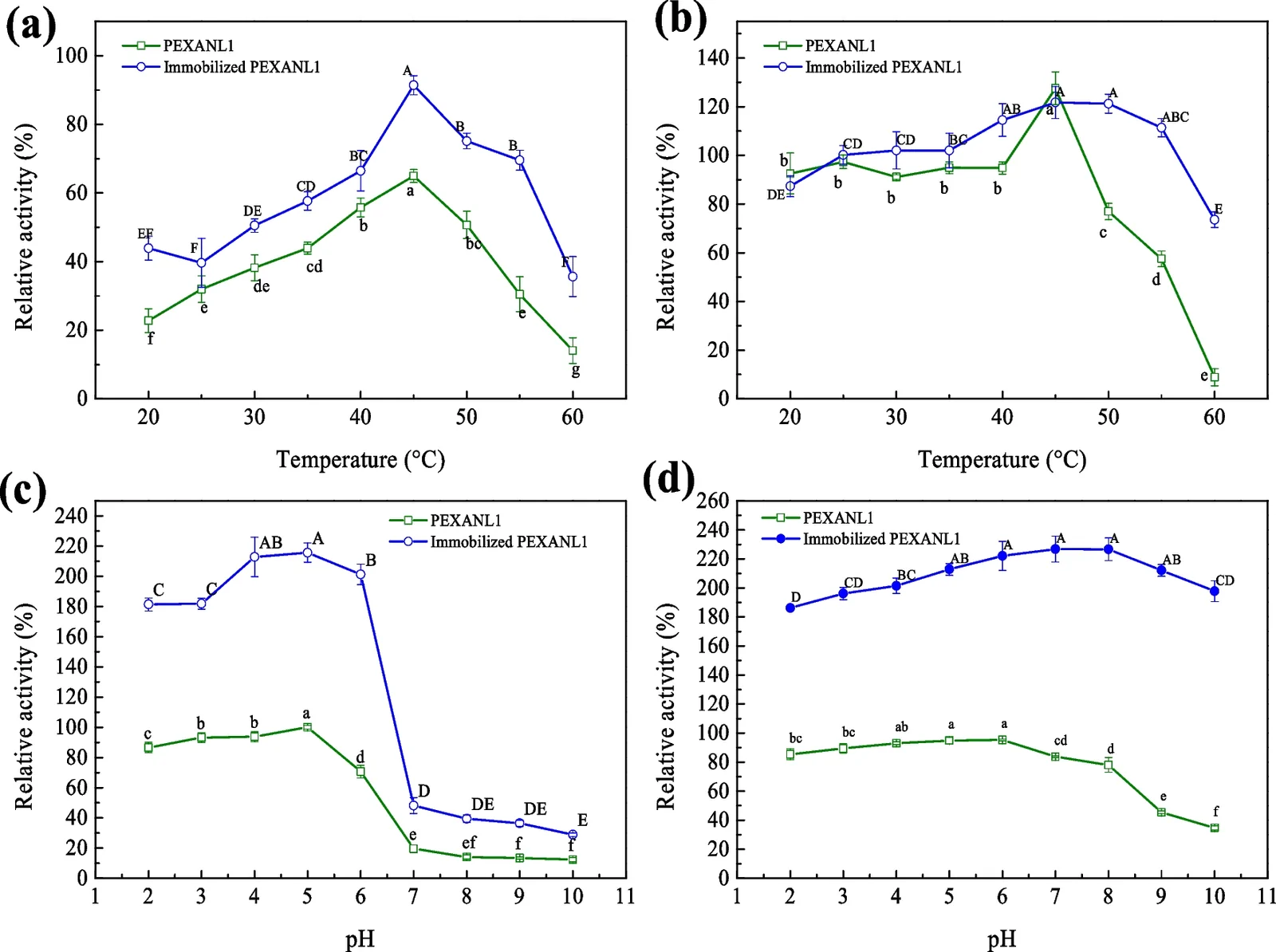
Effect of temperature and pH on PEXANL1 and immobilized PEXANL1. Optimal temperatures of PEXANL1 and immobilized PEXANL1 (**a**); the temperature stability of PEXANL1 and immobilized PEXANL1 (**b**); optimal pH of PEXANL1 and immobilized PEXANL1 (**c**); the pH stability of PEXANL1 and immobilized PEXANL1 (**d**). The enzyme activity was expressed as the relative enzyme activity (%), the enzyme activity measured by PEXANL1 at the optimum temperature (45 °C) and optimum pH (5.0) at protein concentration of 0.2 mg/mL was defined as 100%, corresponding to 3.65 U/mg. The relative enzyme activity under other conditions was calculated based on this. Different lowercase or uppercase letters of the same curve in the same subgraph indicate significant differences (*p* < 0.05)

As shown in Fig. 3(a), the activity of immobilized PEXANL1 initially increased and then decreased with the increase in temperature with the optimum temperature of 45 °C. But, the activity of immobilized PEXANL1 reduced slowly at higher temperatures than that of PEXANL1.

Temperature stability analysis (Fig. 3(b)) revealed that immobilized PEXANL1 retained 73.60% of its original activity after incubating at 60 °C for 1 h. However, the activity of PEXANL1 only remained 8.86% after 1 h incubation at 60 °C.

### Optimum pH and pH stability

As shown in Fig. 3(c), the optimum pH of immobilized PEXANL1 was found to be 5.0. PEXANL1 displayed high activity under acidic conditions (pH 2.0–7.0), with above 80% of its activity, while under alkaline conditions, low activity, less than 30%, was found. Like PEXANL, immobilized PEXANL1 exhibited over 85% of its activity under acidic conditions, while its activity was less than 40% under alkaline conditions.

The pH stability analysis (Fig. 3(d)) revealed that immobilized PEXANL1 retained more than 85% of its original activity at pH 2.0–10.0 after 48 h of incubation, indicating it was stable at pH 2.0–10.0. For PEXANL1, after 48 h of incubation at pH 2.0–8.0, PEXANL1 also kept more than 85% of its original activity, but nearly 50% loss was found after 48 h of incubation at pH 9.0–10.0.

### The operational stability of immobilized PEXANL1

The enzyme activity and operational stability are closely related to the operating costs and economic feasibility associated with the biotransformation process. Therefore, the operational stability of immobilized PEXANL1 was investigated by performing 20 cycles of enzyme reactions. The change in enzyme activity behavior was analyzed by fitting first-order inactivation models into the test data. The first-order inactivation models were estimated by Eq. (4) (Mota et al. 2020):

4 $${A}_{t}={A}_{0}\times {e}^{-{k}_{d}\times t}$$

where *A*_*t*_ is the activity (U/mg) of immobilized PEXANL1 at time *t* (or cycle *n*), *A*_0_ is the initial activity (U/mg), the residual activity (%) of immobilized PEXANL1 at time *t* (or cycle n) was the ratio of *A*_t_ to *A*_0_ multiplied by 100%, and *k*_d_ is the deactivation rate constant. In the present study, *k*_d_ was 0.05226.

The half-life of the immobilized PEXANL1, *t*_1/2_, was estimated by Eq. (5) (Mota et al. 2020):

5 $${t}_{1/2}=\frac{ln2}{{k}_{d}}$$

where *k*_d_ is the deactivation rate constant, and *t* is time (or cycle *n*).

**Fig. 4:**
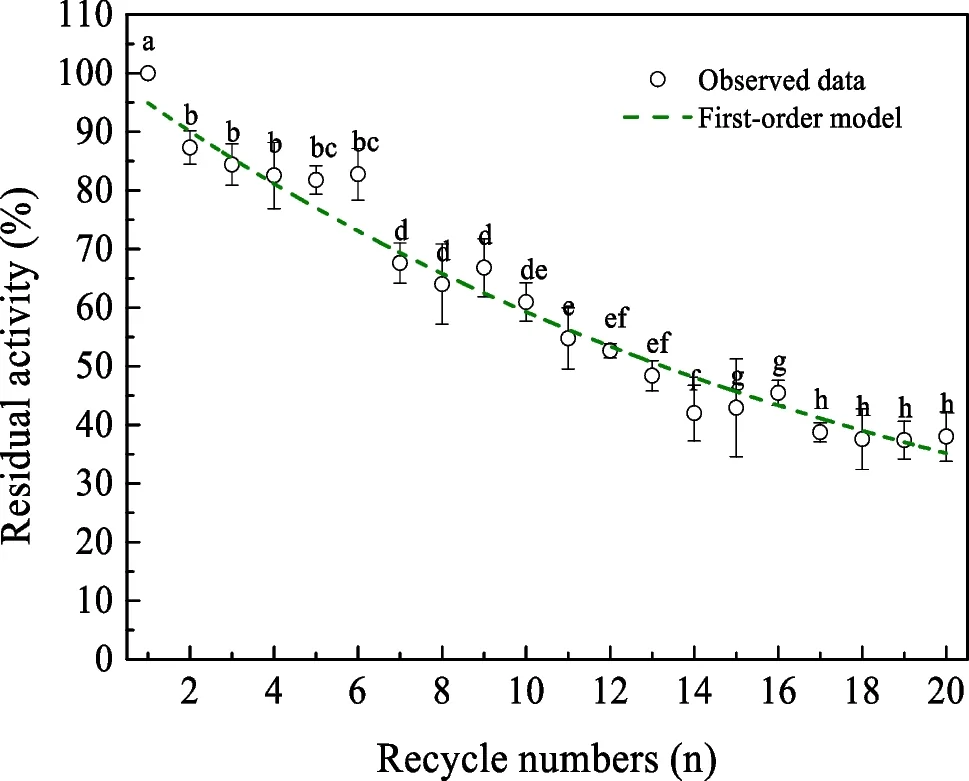
The operational stability of immobilized PEXANL1. The enzyme activity measured by immobilized PEXANL1 at the optimum temperature (45 °C) and optimum pH (5.0) was defined as 100%, corresponding to 7.88 U/mg, that is also the relative activity of cycle 1. Different lowercase letters indicate significant differences (*p* < 0.05)

The reuse of immobilized PEXANL1 is shown in Fig. 4. The retained activity of immobilized PEXANL1 was satisfactory, and the immobilized PEXANL1 retained 86% of its initial activity after 6 consecutive cycles. Although there was more loss of enzyme activity after continued recycling, the retained enzyme activity was still close to 50% after 20 cycles, with a half-life of 13.3 cycles, corresponding to 133 min (approximately 2 h).

### Metal ion tolerance

**Fig. 5:**
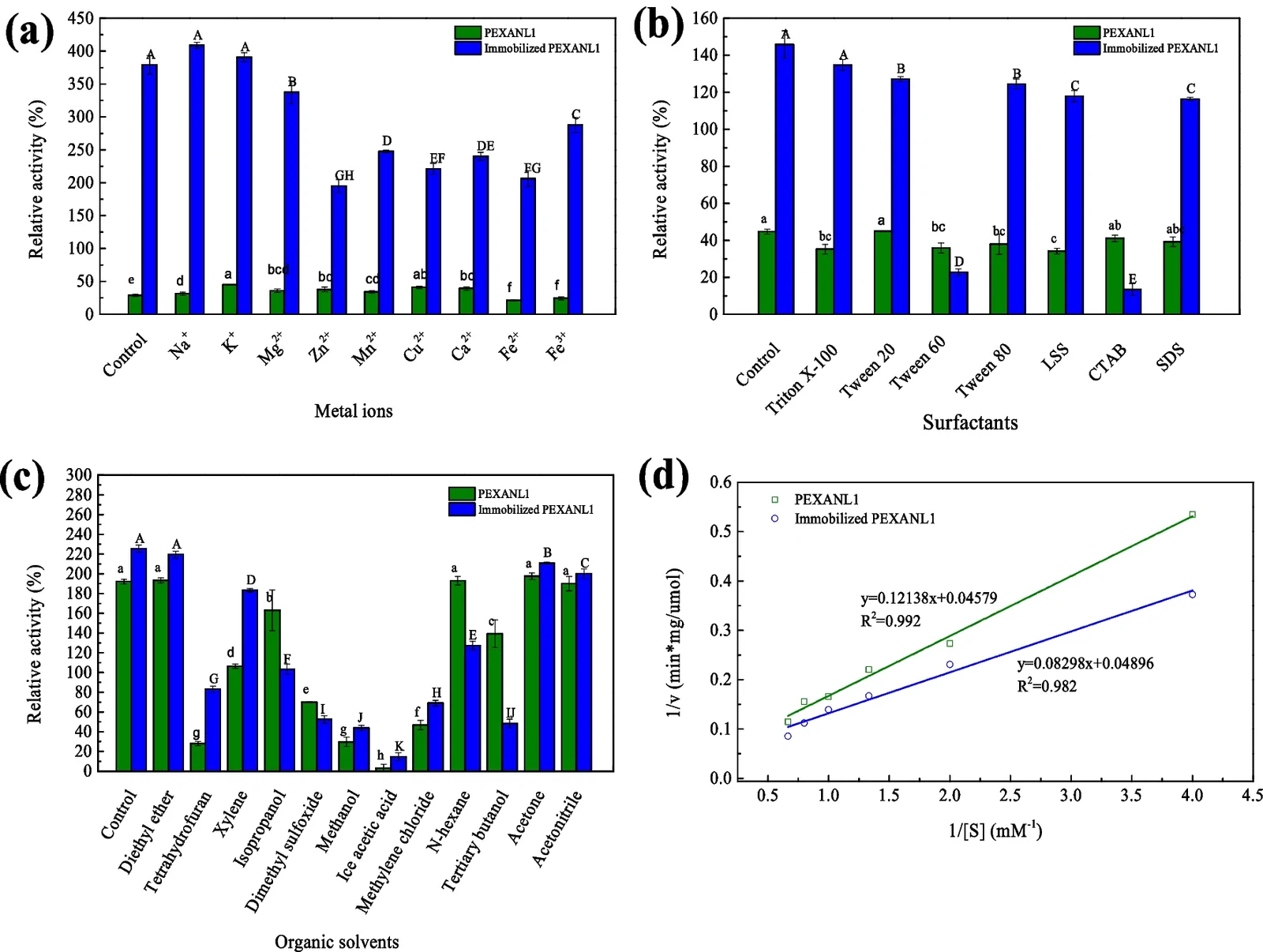
The effect of metal ions (**a**), surfactants (**b**), and organic solvents (**c**) on PEXANL1 and immobilized PEXANL1; kinetic parameters of PEXANL1 and immobilized PEXANL1 (**d**). The enzyme activity was expressed as the relative enzyme activity (%), the enzyme activity measured by PEXANL1 at the optimum temperature (45 °C) and optimum pH (5.0) at protein concentration of 0.2 mg/mL was defined as 100%, corresponding to 3.65 U/mg. The relative enzyme activity under other conditions was calculated based on this. Different lowercase or uppercase letters of the same bar chart in the same subgraph indicate significant differences (*p* < 0.05)

The tolerance to metal ions of PEXANL1 and immobilized PEXANL1 was studied by incubating with different metal ions and EDTA (final concentration 5 mM) at 25 °C for 2 h. As shown in Fig. 5(a), Na^+^, K^+^, Mg^2+^, Zn^2+^, Mn^2+^, Cu^2+^, and Ca^2+^ improved the activity of PEXANL1, while Fe^2+^, Fe^3+^, and EDTA inhibited the activity of PEXANL1. Among them, K^+^ and Cu^2+^ exhibited the highest activation potential for PEXANL1. In contrast, all the metal ions inhibited the activity of immobilized PEXANL1, except Na^+^ and K^+^ (Fig. 5(a)).

### Surfactant and organic solvent tolerance

The effects of non-ionic surfactants (TritonX-100, Tween 20, Tween 60, and Tween 80), cationic surfactants (CTAB), and anionic surfactants (sodium lauryl sarcosinate and SDS) on enzyme activity of PEXANL1 and immobilized PEXANL1 were investigated. As shown in Fig. 5(b), after incubation with kinds of surfactants tested at 25 °C for 2 h, the residual activity of PEXANL1 was 44.49% (Tween 20), 35.17% (TritonX-100), 35.85% (Tween 60), 37.91% (Tween 80), 34.12% (CTAB), 41.13% (sodium lauryl sarcosinate), and 39.22% (SDS), respectively. For immobilized PEXANL1, after incubation with kinds of surfactants tested at 25 °C for 2 h, the residual activity was 127.17% (Tween 20), 134.69% (TritonX-100), 24.86% (Tween 60), 124.49% (Tween 80), 9.37% (CTAB), 117.92% (sodium lauryl sarcosinate), and 116.33% (SDS), respectively.

As shown in Fig. 5(c), the activity of PEXANL1 incubated with ether, hexane, acetone, and acetonitrile was not inhibited, while it was found that tetrahydrofuran, xylene, isopropanol, dimethyl sulfoxide, methanol, and glacial acetic acid inhibited the PEXANL1 activity. Among them, only 27.86% of the relative activity was found after PEXANL1 incubation with tetrahydrofuran, and the activity of PEXANL1 was almost completely lost after incubation with glacial acetic acid. For immobilized PEXANL1, the activity was not strongly inhibited after incubation with ether and acetone, while all other organic solvents inhibited the activity of immobilized PEXANL1. However, the immobilized PEXANL1 improved its tolerance towards xylene, tetrahydrofuran, and dichloromethane compared to the PEXANL1 (Fig. 5(c)).

### Kinetic parameters

The enzyme activity of PEXANL1 and immobilized PEXANL1 within a range of substrate concentrations from 5 to 30 mM under the optimal conditions (45 °C and pH 5.0) was measured. The Lineweaver–Burk plot is shown in Fig. 5(d). The results showed the *K*_m_ of PEXANL1 decreased from 2.65 to 1.69 mmol/L after immobilization. The *V*_max_ of PEXANL1 was slightly reduced from 21.84 to 20.42 µmol/min/mg after immobilization (Table 3).

**Table 3:** Kinetic parameters of lipase before and after immobilization

Biocatalyst	Substrate	*K*_m_ (mM)	*V*_max_ (µmol/min/mg)
PEXANL1	*p*-NPP	2.65	21.84
Fe_3_O_4_@SiO_2_@IL-PEXANL1		1.69	20.42

### The sn-1, 3 specificity

The sn-1, 3 selectivity of PEXANL1 before and after immobilization was determined using the oleic acid and glycerol reaction of ester synthesis, and the content of the products (sn-1, 3-DAG and sn-1, 2-DAG) was measured. The results showed (Table 4) that the sn-1, 3 selectivity of PEXANL1 and immobilized PEXANL1 was 82.35 ± 1.02% and 81.60 ± 0.11%, respectively.

**Table 4:** Changes in regional selectivity before and after immobilization of PEXANL1

Biocatalyst	Sn-1, 3-specificity (%)
PEXANL1	82.35 ± 1.02^a^
Fe_3_O_4_@SiO_2_@IL-PEXANL1	81.60 ± 0.11^a^

The same lowercase letter means the difference was not significant

## Discussion

*A. niger* lipase, as a safe biocatalyst, was widely used in food and oil processing, detergent additive, and other industrial fields. However, *A. niger* lipase has poor stability in environments such as high temperatures, strong alkali, and organic solvents, and it is difficult to recover after the reaction due to its water solubility. In this study, PEXANL1 (an extracellular lipase from *A. niger* GZUF36 heterologously expressed by *P. pastoris* GS115) was immobilized on magnetic nanoparticles modified with ionic liquids. Structure changes of the magnetic nanoparticles during immobilization processes were confirmed by SEM, FT-IR, XRD, VSM, and Zeta potentials. The immobilization conditions were optimized, and the catalytic performance of the immobilized PEXANL1 prepared under the optimal immobilization conditions was characterized.

### SEM, FT-IR, XRD, VSM, and zeta potentials

The size, structure, and morphology of magnetic nanoparticles were analyzed using SEM. Magnetic nanoparticles coated with silica were observed from SEM images (Fig. 1(B(i))). SiO_2_ cladding prevents partial exposure of bare magnetite, thus preventing the accumulation of particles. Subsequently, chloropropyl derivatization was carried out to modify the ionic liquid. Upon immobilizing the enzyme on the ionic liquid-modified magnetic nanoparticles, it was observed that the carrier started to aggregate due to the formation of the enzyme core–shell (Fig. 1(B(v))).

FT-IR was used to determine the changes in functional groups of magnetic nanoparticles. Through FT-IR, the composition of the carrier was confirmed. In addition, the enzyme fixed on the carrier was also identified.

VSM is used to measure the magnetization intensity of magnetic nanoparticles. The saturation magnetization of immobilized PEXANL1 was lower than that of Fe_3_O_4_@SiO_2_@ILs (Fig. 1(E)), caused by the adsorption of enzymes on nanocapsules through electrostatic interaction. Interestingly, although the magnetic strength of the immobilized enzyme decreased, the magnetic strength of the magnetically immobilized enzyme prepared in this study was higher than that of some scholars (Defaei et al. 2018). On the one hand, the magnetic immobilized PEXANL1 prepared in this study was superior. On the other hand, using a magnet to recover the immobilized PEXANL1 was beneficial.

The zeta potentials were used to analyze the electrostatic potential of magnetic nanoparticle suspensions very close to the surface. PEXANL1 was negatively charged in the buffer system at pH 7.0 through zeta potential analysis, and Fe_3_O_4_ was negatively charged in the same system. Fe_3_O_4_@SiO_2_ was much larger than that of Fe_3_O_4_, indicating that the surface modification of Fe_3_O_4_ with SiO_2_ reduced aggregation of Fe_3_O_4_; that is, the magnetic carrier was more stable, and the silica was chemically stable and capable of various surface modifications. The zeta potential values of Fe_3_O_4_@SiO_2_@CP-TMS were significantly increased after the chloropropyl derivatization of Fe_3_O_4_@SiO_2_, and the suitable alkyltrimethoxysilane-modified magnetic nanoparticles are ideal carriers for lipase immobilization (Wang et al. 2015). Khoshnevisan’s study also showed that the bare surface nanoparticles were negatively charged at higher pH (pH > 6.0). In comparison, the coated nanoparticles were negatively charged at lower pH (pH < 6.0) due to the coordination of iron atoms on the surface of magnetic particles with hydroxyl ions or H_2_O, which usually dissociate to form a negative charge covered by hydroxyl groups (Khoshnevisan et al. 2016). The Fe_3_O_4_@SiO_2_@ILs was positively charged, which was consistent with the findings that imidazole cations in ionic liquids could form complexes with negatively charged surfaces (Jiang et al. 2009), but the stability of Fe_3_O_4_@SiO_2_@ILs was lower. The zeta potential analysis also indicated an increase in the stability of PEXANL1 immobilized onto Fe_3_O_4_@SiO_2_@ILs.

### Optimizing conditions for immobilized PEXANL1

Many factors influence immobilization efficiency and enzyme activity recovery of immobilized PEXANL1, such as anionic surfactant type and content, pH, carrier addition content, immobilization time, and enzyme concentration. For the anionic surfactant type, SDS was selected as the suitable surface modifier (Fig. 2(a)). SDS was a medium to immobilize lipase onto Fe_3_O_4_@SiO_2_@ILs via hydrophobic interactions. However, the amount of SDS reduced the activity recovery of immobilized PEXANL1 (Fig. 2(b)). It is speculated to be due to SDS binding to the active center and inhibiting substrate entry (Zhang et al. 2018). In addition, SDS can also lead to lipase denaturation (Fano et al. 2011; Rasmussen et al. 2022).

For pH, it was observed that the optimal immobilization pH was 6.0 (Fig. 2(c)). There were hydrophobic interactions and electrostatic interactions between PEXANL1 and Fe_3_O_4_@SiO_2_@ILs under the immobilization process. The electrostatic and hydrophobic interactions between the enzyme and the immobilized carrier are critical for the enzyme to achieve high loading density through adsorption (Min et al. 2012). However, if PEXANL1 and Fe_3_O_4_@SiO_2_@ILs in the buffer were positive charges, adsorption capacity would decrease due to electrostatic repulsive. In pH 6.0, PEXANL1 and Fe_3_O_4_@SiO_2_@ILs had opposite charges, simultaneously electrostatic and hydrophobic interactions, and thus the maximum activity recovery was obtained.

Enzyme concentration is an important factor affecting immobilization. Generally, the more enzyme was added, the higher the activity recovery. However, higher enzyme concentration can lead to decreased activity recovery. It was evidence that there was no significant difference in immobilization efficiency within the enzyme protein concentration range of 0.3–0.5 mg/mL (Fig. 2(f), *p* < 0.05). Therefore, it can be inferred that the carrier adsorption capacity has begun to reach saturation when the enzyme concentration is 0.3 mg/mL. Moreover, too many enzymes can lead to enzyme aggregation, pore crowding, or the formation of multilayers on the carrier surface at the same size (Sigurdardóttir et al. 2018).

Finally, the single-factor experiment obtained the optimal activity recovery and immobilization efficiency of immobilized PEXANL1 when SDS concentration (w/v) was 0.015, pH was 6.0, carrier addition amount was 15 mg, immobilization time was 3 h, and the enzyme concentration was 0.3 mg/mL (Table 2).

### Performance evaluation of immobilized PEXANL1

Immobilization technology can improve the enzyme’s ability to withstand high-temperature production and operation conditions. Here, the optimum temperature and temperature stability of immobilized PEXANL1 were investigated. The optimal temperature of immobilized PEXANL1 was 45 °C, the same as that of PEXANL1 (Fig. 3(a)), suggesting the optimum temperature did not change after immobilization. Temperature stability analysis revealed immobilized PEXANL1 maintained higher activity at elevated temperatures than PEXANL1 (Fig. 3(b)), implying an enhancement in thermal stability of PEXANL1 after immobilization. The increased thermal stability of immobilized PEXANL1 was consistent with the reports (Abdulhamid et al. 2021; Bezerra et al. 2020; Zhang et al. 2020; Zhu et al. 2021). Immobilization technology is commonly believed to enhance the enzyme structure’s rigidity and improve thermal stability (Ismail and Baek 2020).

The pH stability of immobilized PEXANL1 was significantly improved under acidic conditions (pH 2.0 ~ 7.0) (Fig. 3(d)). Also, compared to PEXANL1, it was observed that immobilized PEXANL1 exhibited higher activity after incubation for 48 h at pH 9.0–10.0 (Fig. 3(d)). These findings indicated that immobilized PEXANL1 broadened the pH tolerance range (from pH 2.0–8.0 to pH 2.0–10.0). So, immobilized PEXANL1 with this property will be potentially helpful in the wastewater treatment industry.

Operational stability assessment revealed that immobilized PEXANL1 retained 86% after 6 cycles, with a half-life of 13.3 cycles, equivalent to 133 min (approximately 2 h). The robust electrostatic and hydrophobic interactions between PEXANL1 and Fe_3_O_4_@SiO_2_@ILs contributed to the high operational stability of immobilized PEXANL1. The observed decline in enzyme activity during continuous recycling (Fig. 4) could be attributed to the repeated separation from the reaction system, which may lead to a reduction in the quality of the immobilized enzyme and to the mechanical damage incurred through its repeated use (Carvalho de Melo et al. 2023).

Metal ion tolerance tests revealed the improved activity of immobilized PEXANL1 in Na^+^, Ca^2+^, and K^+^. It could be attributed to ionic interactions between the negatively charged groups on the PEXANL1 surface and metal ions, which enhance substrate affinity. Ca^2+^ may help the lipase lid open to expose the active center for substrate entry and binding (Kuwahara et al. 2008).

Surfactant tolerance tests revealed immobilized PEXANL1 tolerated these tested surfactants, especially Tween 60 and CTAB. Solvent tolerance assessments demonstrated the enhanced activity and stability of immobilized PEXANL1 in xylene, tetrahydrofuran, and dichloromethane. It is likely attributable to increased enzyme hydrophobicity after ionic liquid modification. Notably, in hydrolysis and esterification reactions, hydrophobic ionic liquids enhance the performance of immobilized lipases (Jafari et al. 2020; Lee et al. 2007). Generally, most natural enzymes are prone to denaturation and inactivation when exposed to organic solvents (Ogino and Ishikawa 2001). Therefore, immobilized PEXANL1 may broaden its industrial applications due to enhanced resistance to surfactants and organic solvents.

The enzyme–substrate kinetic analysis revealed that the substrate affinity of PEXANL1 became higher after immobilization (Table 4). The hydrophobic nature of the ionic liquid-modified magnetic carrier prompted the lipase to adopt an open-cap conformation. Its change increased the size of the local entrance pore of the lipase, exposing more active sites, making it easier for the substrate to bind to the immobilized enzyme (Xu et al. 2022), and thus the increased substrate affinity.

Lipases specific to sn-1, 3 positions hold significant potential for synthesizing structured oils (Cui et al. 2022). The sn-1, 3 specificity analysis indicated that immobilization did not alter the sn-1, 3 selectivity of PEXANL1 (Table 4, *p* > 0.05), aligning with findings documented in the literature (Cui et al. 2022; Zhu et al. 2021).

Here, we synthesized a suitable ionic liquid-modified magnetic nanocarrier (Fe_3_O_4_@SiO_2_@ILs) functionalized using highly active and stable surfactants. Ionic liquid-modified magnetic nanocarrier could be used to immobilize PEXANL1 via electrostatic and hydrophobic interactions to improve its pH, metal ion, surfactants, and organic solvent stability. The immobilization conditions were optimized, and the specific activity of the immobilization enzyme was more than twice that of the free enzyme. SEM, FT-IR, XRD, VSM, and Zeta potential demonstrated the attachment of PEXANL1 to Fe_3_O_4_@SiO_2_@ILs. In addition, due to the magnetic properties of the synthetic nanomaterials, the immobilized PEXANL1 could be easily separated from the reaction mixture and reused in the next round of reaction. In the hydrolysis reaction of *p*-NPP, the immobilized PEXANL1 was used 6 times, the remaining activity was still 86%, and the half-life was 13.3 cycles, corresponding to 133 min (about 2 h). This study indicated that PEXANL1, immobilized by this strategy, exhibited high and good operational stability. Therefore, we believe that in some cases, ionic liquid-modified magnetic nanomaterials may also be a promising nanomaterial suitable for other industrial enzymes.

## Data Availability

All data generated or analyzed during this study are included in this published article.
